# Gain-of-Function Properties of a Dynamin 2 Mutant Implicated in Charcot-Marie-Tooth Disease

**DOI:** 10.3389/fncel.2021.745940

**Published:** 2021-10-20

**Authors:** Tara C. Tassin, Barbara Barylko, Per Niklas Hedde, Yan Chen, Derk D. Binns, Nicholas G. James, Joachim D. Mueller, David M. Jameson, Ronald Taussig, Joseph P. Albanesi

**Affiliations:** ^1^Department of Pharmacology, U.T. Southwestern Medical Center, Dallas, TX, United States; ^2^Department of Cell and Molecular Biology, John A. Burns School of Medicine, University of Hawaii, Honolulu, HI, United States; ^3^Laboratory for Fluorescence Dynamics, University of California, Irvine, Irvine, CA, United States; ^4^School of Physics and Astronomy, University of Minnesota, Minneapolis, MN, United States

**Keywords:** dynamin, Charcot-Marie-Tooth disease, centronuclear myopathy, tyrosine phosphorylation, FLIM/FRET, fluorescence fluctuation spectroscopy

## Abstract

Mutations in the gene encoding dynamin 2 (DNM2), a GTPase that catalyzes membrane constriction and fission, are associated with two autosomal-dominant motor disorders, Charcot-Marie-Tooth disease (CMT) and centronuclear myopathy (CNM), which affect nerve and muscle, respectively. Many of these mutations affect the pleckstrin homology domain of DNM2, yet there is almost no overlap between the sets of mutations that cause CMT or CNM. A subset of CMT-linked mutations inhibit the interaction of DNM2 with phosphatidylinositol (4,5) bisphosphate, which is essential for DNM2 function in endocytosis. In contrast, CNM-linked mutations inhibit intramolecular interactions that normally suppress dynamin self-assembly and GTPase activation. Hence, CNM-linked DNM2 mutants form abnormally stable polymers and express enhanced assembly-dependent GTPase activation. These distinct effects of CMT and CNM mutations are consistent with current findings that DNM2-dependent CMT and CNM are loss-of-function and gain-of-function diseases, respectively. In this study, we present evidence that at least one CMT-causing DNM2 mutant (ΔDEE; lacking residues ^555^DEE^557^) forms polymers that, like the CNM mutants, are resistant to disassembly and display enhanced GTPase activation. We further show that the ΔDEE mutant undergoes 2-3-fold higher levels of tyrosine phosphorylation than wild-type DNM2. These results suggest that molecular mechanisms underlying the absence of pathogenic overlap between DNM2-dependent CMT and CNM should be re-examined.

## Introduction

Dynamin 2 (DNM2) is a ubiquitously expressed ∼94 kDa force-generating enzyme that forms helical polymers around the necks of budding vesicles and couples GTP hydrolysis to the catalysis of membrane scission (Antonny et al., 2016). In addition to DNM2, mammalian cells express two other forms of dynamin (DNM1 and DNM3), with more restricted subcellular distributions. The three mammalian dynamins share a common domain structure, including an N-terminal GTPase domain, a “middle domain” implicated in self-association, a pleckstrin homology domain (PHD) that mediates binding to phosphatidylinositol 4,5-bisphosphate (PIP_2_) in the plasma membrane, a GTPase effector domain (GED) that folds back to interact with both the middle and catalytic domains, and a C-terminal proline/arginine rich domain (PRD) that interacts with numerous SH3 domain-containing proteins. Together, the three α-helices of the middle domain and the single α-helix of the GED form an antiparallel four-helix bundle termed the “stalk.” Crystal structures of DNM1 (Faelber et al., 2011; Ford et al., 2011) and DNM3 (Reubold et al., 2015) indicate that stalks from adjacent dynamins form interfaces that drive dynamin self-assembly. These structural studies also revealed that, in unassembled dynamins, the PHD is folded back onto the stalk, inducing an auto-inhibited “closed” conformation that prevents oligomerization beyond the tetrameric state. Binding of the PHDs to PIP_2_-containing membranes displaces them from the stalk and induces an “open” active dynamin conformation capable of self-assembly (Mears et al., 2007; Srinivasan et al., 2016) (see Figure 2A). Although the interaction of dynamins with PIP_2_ is essential for their function in endocytosis (Achiriloaie et al., 1999; Anthony et al., 1999; Valus et al., 1999), it is not required for targeting them to endocytic sites on the plasma membrane, which instead involves interactions between dynamin PRDs and SH3 domains of endocytic co-factors (Bethoney et al., 2009). Therefore, the major role of the PHD-PIP_2_ interaction is likely to be induction of the closed-to-open conformational switch.

Mutations in the DNM2 gene cause two autosomal dominant motor disorders, centronuclear myopathy (CNM) (Bitoun et al., 2005) and Charcot-Marie-Tooth disease (CMT) (Züchner et al., 2005), which affect muscle and nerve, respectively (Zhao et al., 2018). Although the majority of residues affected by these mutations are located in the PHD, there is almost no overlap between mutations that cause CNM and CMT, suggesting that the two disorders do not share common molecular or pathogenic mechanisms. CNM mutations examined to date have been reported to enhance DNM2 self-assembly and GTPase activity without affecting PIP_2_ binding (Kenniston and Lemmon, 2010; Wang et al., 2010). In contrast, CMT mutations have been found to inhibit PIP_2_ binding and PIP_2_-stimulated GTPase activity (Kenniston and Lemmon, 2010; Reubold et al., 2015). These phenotypes were explained by x-ray structural analyses, which revealed that most CNM mutations are located in the PHD-stalk interface, whereas most CMT mutations are clustered in the PIP_2_-binding pocket of the PHD. Thus, CNM mutations are likely to disrupt the closed, auto-inhibited DNM2 conformation, while CMT mutations are likely to increase auto-inhibition by suppressing the PHD-PIP_2_ interaction (Faelber et al., 2013). These *in vitro* observations have been cited to support the currently prevailing view that DNM2-associated CNM and CMT are gain-of-function and loss-of-function disorders, respectively (Chin et al., 2015; Cowling et al., 2017; Buono et al., 2018; Massana Muñoz et al., 2019; Zhao et al., 2019). However, we show in this study that a CMT-causing DNM2 mutant that lacks three acidic residues (^555^DEE^557^) within the PHD forms more stable polymers, expresses higher assembly-dependent GTPase activity, and undergoes higher levels of tyrosine phosphorylation than wild-type (WT) DNM2. Thus, our data challenge the widely held assumption that all CNM-linked mutants are hyperactive, whereas all CMT-linked mutants are hypoactive.

## Experimental Methods

### Reagents

Ni-NTA was from Qiagen (Valencia, CA). IPL-41 and pluronic F-68 for growing Sf9 cells were from Invitrogen (Carlsbad, CA). Mutagenesis primers were from Integrated DNA Technologies (Coralville, IA). Cloning and mutagenesis reagents, Protein A/G-Sepharose, and FastAP alkaline phosphatase were from Thermo Scientific. Anti-FLAG affinity resin (M8823), 3X FLAG peptide (F4799), and anti-FLAG antibody (F7425) were from Sigma. Anti-Src antibody was from Santa Cruz (sc-18). Anti-phosphotyrosine 4G10 Platinum antibody was from Millipore. Phosphatidylinositol 4,5-bisphosphate (PIP_2_) and brominated phosphatidylcholine (PC), were from Avanti Polar Lipids. [γ-^32^P]GTP and [γ-^32^P]ATP were from PerkinElmer Life Sciences. Reagents for electrophoresis and immunoblotting were from Bio-Rad. Fluorescently labeled secondary antibodies for Infrared Imaging System were from LICOR. Other reagents, including anti-myc antibodies, buffers, charcoal-activated Norit A, GSH-Agarose, glutathione, ATP, GTP, protease inhibitors and PMSF were from Sigma (St. Louis MO). pCMV5 mouse Src was from Addgene (plasmid #13663, provided by Joan Brugge and Peter Howley).

### Purification of Dynamin 2 Mutants

Mutations were introduced into rat DNM2 isoform 1 (also known as isoform “ba” or “IIBA”; UniProtKB identifier P39052-1) using the QuikChange site-directed mutagenesis kit (Stratagene). cDNAs encoding wild-type DNM2 (DNM2^WT^) and the DNM2^K562E^ and DNM2^A618T^ mutants containing C-terminal His_6_ tags were subcloned into BacPAK9 plasmids (Clontech) and purified as described previously (Lin et al., 1997). The DNM2^Δ^ ^DEE^ mutant was generated with a C-terminal FLAG tag in a Fast-Bac vector. To purify this construct, infected Sf9 cells were homogenized in buffer A (20 mM HEPES (pH 7.5), 300 mM NaCl, 3 mM MgCl_2_, 0.5 mM DTT, 1 mM EDTA, and 0.2 mM PMSF) supplemented with a protease inhibitor cocktail consisting of 10 μg/ml each of N-p-tosyl-L-lysine chloromethyl ester, N-p-tosyl-L-arginine methyl ester, N-p-tosyl-L-lysine chloromethyl ketone, leupeptin, and pepstatin A (without phosphatase inhibitors). The homogenate was centrifuged, and the supernatant was mixed with FLAG resin for 1.5 h, washed, and eluted with 3x-FLAG peptide. Purified dynamins were dialyzed against buffer A. Aliquots of purified proteins were frozen in liquid nitrogen and stored at −70°C. Immediately before use, the dynamin solutions were centrifuged at 213,000 × *g* for 15 min to remove aggregates.

### Purification of Dynamin 2 Pleckstrin Homology Domains

Rat DNM2 PHD, comprising residues 510–620, was subcloned into a pGEX-KG vector using full length DNM2 as a template. GST-PHD was expressed in BL21 *E. coli* cultured for 4 h at 37°C in the presence of 0.5 mM IPTG. Cells were lysed in PHD-lysis buffer (20 mM HEPES (pH 7.5), 100 mM NaCl, 1 mM BME, 0.2 mM PMSF, and the protease inhibitor cocktail described above, and cell suspensions were centrifuged at 35,000 rpm in a Ti45 rotor for 30 min at 4°C. Supernatants were incubated for 1 h at 4°C with GSH-agarose beads, the beads were washed in PHD-lysis buffer containing 1 M NaCl, then eluted with PHD-lysis buffer containing 15 mM glutathione. Eluted protein was dialyzed overnight in lysis buffer. Mutations were introduced using the QuikChange mutagenesis kit using the WT PHD as template.

### Preparation of Dynamin Constructs in Mammalian Cells

Constructs were generated by cloning dynamin cDNAs into vectors containing FLAG, myc, or EGFP tags for C-terminal positioning. cDNAs encoding wild-type DNM2 (DNM2^WT^) and the DNM2^K562E^, and DNM2^A618T^ mutants, all with C-terminal His_6_ tags, were used as templates. The DNM2^Δ^ ^DEE^ mutant was generated by introducing the deletion mutation into FLAG, myc, or EGFP-tagged DNM2^WT^ templates using the QuikChange site-directed mutagenesis kit (Stratagene).

### GTPase Assays

GTPase activities were measured by the release of ^32^P_i_ from [γ-^32^P]GTP following incubation at 37°C. Assay solutions contained 20 mM HEPES (pH 7.5), 2 mM MgCl_2_, 1 mM GTP, and 100 mM NaCl. Reactions were initiated by addition of MgGTP and terminated by addition of 5% Norit-A activated charcoal in 50 mM NaH_2_PO_4_ at 4°C (Higashijima et al., 1987). All procedures involving radioactivity were carried out using protocols approved by the UT Southwestern Office of Safety and Business Continuity.

### Turbidity Assays

Wild-type (WT) and mutant forms of DNM2 in 300 mM NaCl were placed in quartz cuvettes and diluted with pre-warmed (37°C) 20 mM HEPES (pH 7.5) to obtain final concentrations of 1 μM dynamin and 50 mM NaCl. Self-assembly was monitored by measuring absorbance at 350 nm at 37°C and 15 s increments using a Beckman DU 650 spectrophotometer.

### Liposome Binding Assay

Liposome binding was performed by sedimentation assay according to Lee and Lemmon (Lee and Lemmon, 2001) using a brominated-PC:PIP_2_ molar ratio of 97:3. Lipids were dissolved in chloroform, then dried under a stream of nitrogen followed by overnight drying under vacuum. Dried lipids were resuspended in 20 mM HEPES (pH 7.4) and 100 mM NaCl, followed by 10 freeze/thaw cycles in liquid nitrogen and bath sonication. To extract unilamellar vesicles and remove aggregated lipids, liposomes were extruded 10 times through 0.1 μm filters using a Mini–Extruder (Avanti Polar Lipids). Immediately prior to incubation with liposomes, solutions containing PHDs were centrifuged for 1 h at 265,000 × *g* at 25°C in a TL-100 tabletop ultracentrifuge. Binding assays were carried out by incubating 5 μM PHD with liposomes for 15 min at 25°C in 20 mM HEPES (pH 7.4) and 50 mM NaCl. Samples (80 μl) were then centrifuged at 214,000 × *g* for 1 h at 25°C. Supernatants were removed and pellets were resuspended in initial sample volumes. Equal volumes of samples before centrifugation and resuspended pellets were electrophoresed, proteins were visualized by Coomassie blue staining and quantified by LiCOR Odyssey system scanning.

### Brightness Analysis

Brightness analysis relies on the fact that mobile proteins with multiple attached fluorophores (e.g., tetramers) may be distinguished from those containing a single fluorophore (monomer) by analysis of the fluctuations of the average intensity. This principle is illustrated in Supplementary Figure 1. We note that the method requires comparison of the fluctuations, taken under identical instrument settings, from a monomeric brightness standard – in our case EGFP. Hence, by normalizing the measured brightness with the brightness of the monomer standard, the oligomerization state of our target protein can be quantified. The instrumentation for brightness analysis fluorescence fluctuation measurements was described previously (Chen et al., 2003). An excitation wavelength of 1,000 nm (for two-photon excitation) was used for all experiments. Brightness is calculated with Q-analysis (Sanchez-Andres et al., 2005). Monomer brightness of EGFP was obtained by averaging over 5 cells at various concentrations. The resulting normalized molecular brightness, *b*, is calculated by taking the brightness of an individual measurement divided by the brightness of monomeric EGFP.

### Raster Scan Image Correlation Spectroscopy

For Raster Scan Image Correlation Spectroscopy (RICS) analysis, NIH3T3 cells plated on fibronectin-coated imaging dishes were transfected with plasmids encoding EGFP-tagged dynamins using Lipofectamine 3000. Cells were imaged 20–26 h after transfection at room temperature for a maximum duration of 90 min with an Olympus FV1000 confocal microscope set up for RICS. Fluorescence was excited with 488-nm light and green fluorescence was detected in a band of 505–525 nm with a 60×, NA 1.2 water immersion lens. For each RICS data set, 100 frames of 256 × 256 pixels were acquired with a pixel dwell time of 10 μs and a line time of 3.68 ms, pixel size was 50 nm, the waist (w_0_) of the point spread function was 270 nm. Before raster image correlation, a moving average of 2 frames was subtracted to remove the immobile fraction. Data were analyzed using SimFCS software.

### Dynamin Phosphorylation

To monitor DNM2 phosphorylation in cells, HeLa cells transfected with Lipofectamine-2000 were incubated with 0.1 mM sodium orthovanadate for 1 h before lysis in RIPA buffer (50 mM Tris, pH 8.0; 150 mM NaCl, 1% NP-40, 0.5% sodium deoxycholate, 5 mM EDTA, 0.1% SDS, 50 mM glycerophosphate) containing protease inhibitor cocktail (see above) and 1 tablet/10 ml buffer of Phosphatase Inhibitor Cocktail Tablets (Roche). Lysates were centrifuged at 1000 × *g* for 10 min at 4°C and resulting supernatants were incubated for 4 h with anti-FLAG antibody conjugated to agarose beads or anti-myc antibodies followed by protein A/G Sepharose to immunoprecipitate dynamins. Samples were washed 3 times with RIPA buffer supplemented with protease and phosphatase inhibitors (as above), then electrophoresed and blotted with anti-FLAG or anti-myc and anti-phosphotyrosine (4G10) antibodies. Immunoblots were scanned and quantified by LiCOR Odyssey. To phosphorylate DNM2 *in vitro*, FLAG tagged DNM (WT or mutant) were expressed in HEK293 cells and immunoprecipitated with anti-FLAG antibody conjugated to agarose beads in the absence of SDS or phosphatase inhibitors. To obtain non-phosphorylated dynamin, the beads were additionally incubated overnight at 4°C with 15 units/ml FastAP alkaline phosphatase (AP). The beads were then washed extensively with a solution containing 20 mM HEPES, pH 7.5, 150 mM NaCl, and 0.1% Triton to remove AP, and incubated with v-Src in the presence of 10 mM MgCl_2_ and 0.5 mM ATP. Samples were electrophoresed, blotted with anti-FLAG and anti-phosphotyrosine antibodies, and scanned and quantified by LiCOR Odyssey.

### Other Methods

Cells were grown in Dulbecco’s modified Eagle’s medium containing 10% fetal calf serum and antibiotics at 37°C in 5% CO_2_. Protein concentration was determined using the modified Lowry method (Lowry et al., 1951) according to Peterson (Peterson, 1977) with BSA as a standard. SDS-PAGE was carried out according to Laemmli (Laemmli, 1970).

## Results

### The ΔDEE Mutation Does Not Affect the PIP_2_ Binding by the Dynamin 2 Pleckstrin Homology Domain

**FIGURE 1:**
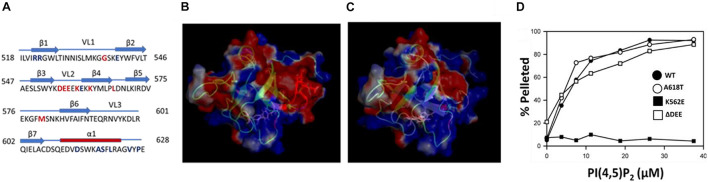
The ΔDEE mutation does not affect binding of the DNM2 PHD to PIP_2_. **(A)** Location of the ^555^DEE^557^ motif within VL2 of the PHD. Residues mutated in CMT and CNM are in red and blue, respectively. **(B)** Electrostatic potential surface of the DNM2 PHD. Red indicates negative potential; blue indicates positive potential. Residues ^555^DEE^557^ are shown in red (modified from DNM2 PHD, PDB ID:2ys1 (Li et al., 2008). **(C)** Electrostatic potential surface of the DNM2^Δ^ ^DEE^ PHD. **(D)** Binding of isolated WT and mutant DNM2 PHDs (5 μM each) to PIP_2_-containing vesicles. A co-sedimentation assay (Lee and Lemmon) was used to measure binding of purified GST-PHDs to small unilamellar vesicles composed of brominated-PC:PIP_2_ at a molar ratio of 97:3.

Lysines that contribute to the interaction of dynamins with PIP_2_ are clustered in variable loop 2 (VL2) and β-strand 4 (β4) of the PHD (Figure 1A). The best studied CMT mutation in DNM2, K562E, is located in strand β4 and essentially eliminates PIP_2_ binding and GTPase activation (Kenniston and Lemmon, 2010). The CMT-linked mutant examined in this study, DNM2^Δ^ ^DEE^, contains a deletion of residues ^555^DEE^557^ in VL2. This deletion increases the electrostatic potential of VL2 (Figures 1B,C), potentially enhancing the association of the PHD with the negatively charged PIP_2_ headgroup. However, we found that the ΔDEE mutation did not alter PHD binding to small unilamellar vesicles composed of phosphatidylcholine (PC) and PIP_2_ (97:3 molar ratio) (Figure 1D). As expected, the K562E mutation prevented PHD binding to PC/PIP_2_ vesicles, whereas the A618T CNM mutation had no effect.

**FIGURE 2:**
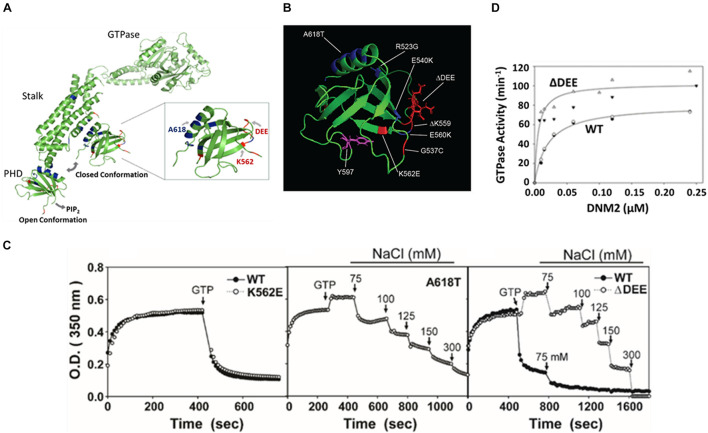
The ΔDEE mutation stabilizes DNM polymers and enhances assemb*ly-dependent* GTPase activation. **(A)** Illustration of different placements of PHD in extended and closed dynamin conformations. The sites of the mutations examined in this study are highlighted. K562E and deletion of DEE are CMT-linked mutations. A618T is a CNM-linked mutation (modified from Dynamin 1 tetrameric cryoEM structure, PDB ID: 6DLV (Kong et al., 2018). Note that the PRD was removed for crystallographic *analyses*. **(B)** Location of CMT-linked mutations (red) and CNM-linked mutations (blue) in the DNM2 PHD. Tyrosine 597, phosphorylatable by Src kinase, is shown in purple (modified from DNM2 PHD, PDB ID:2ys1 (Li et al., 2008). **(C)** Stabilization of dynamin 2 ΔDEE mutant DNM2 polymers. Solutions containing DNM2^WT^, CNM-linked mutant DNM2^A618T^ or CMT-linked mutants DNM2^Δ^ ^DEE^ and DNM2^K562E^ at a final concentration of 1 μM were induced to polymerize by reduction of NaCl concentration from 300 to 50 mM. After reaching plateaus, GTP and MgCl_2_ were added to final concentrations of 1 and 2 mM, respectively. Arrows indicate addition of GTP and MgCl_2_ or of NaCl to achieve designated final concentrations. **(D)** GTPase activation of DNM2^WT^ and DNM2^Δ^ ^DEE^ as a function of dynamin concentration. Open and filled triangles represent data obtained from two separate preparations of DNM^Δ^ ^DEE^. Open diamonds and filled circles represent data obtained from two separate preparations of DNM2^WT^.

### Stabilization of Dynamin 2 Polymers by Deletion of ^555^DEE^557^

At low ionic strength, dynamins self-assemble *in vitro* into rings and coils (Hinshaw and Schmid, 1995) resembling those that form around the necks of budding vesicles (Van der Bliek et al., 1993; Takei et al., 1995). Self-assembly stimulates dynamin GTPase activities from basal levels of ∼1–10 min^–1^ to activated levels of ∼100–200 min^–1^. Disassembly of *in vitro*-formed dynamin polymers occurs rapidly upon increase of ionic strength or addition of GTP (Warnock et al., 1996), presumably reflecting the GTPase-associated disassembly of dynamin rings that accompanies membrane scission in cells. In a prior study, we found that introduction of CNM-linked mutations R365W, E368K, and A618T stabilizes DNM2 polymers against GTP- and salt-induced disassembly (Wang et al., 2010). Structural studies suggest that the mutations weaken the PHD-stalk interface and, hence, would favor the open, assembly competent DNM2 conformation. Like most other residues mutated in CMT, the ^555^DEE^557^ motif is located on the opposite surface of the PHD to the C-terminal helix that contributes to the PHD-stalk interface (Figures 2A,B). Therefore, we were surprised to find that DNM2^Δ^ ^DEE^ showed almost identical resistance to GTP- and salt-induced disassembly as DNM2^A618T^ (Figure 2C). In contrast, the assembly properties of the CMT mutant DNM2^K562E^ were similar to those of DNM2^WT^. Consistent with the enhanced self-assembly induced by the ΔDEE mutation, GTPase activation of DNM2^Δ^ ^DEE^ occurred at lower DNM2 concentrations and displayed higher maximal levels than DNM2^WT^ (Figure 2D). These results demonstrate that at least one CMT-linked mutation, ΔDEE, confers the gain-of-function *in vitro* properties that were hitherto observed only with CNM-linked mutations, and raise the possibility that the simple “stalk-occlusion” mechanism for auto-inhibition of dynamin polymerization by the PHD may be an oversimplification.

### The ΔDEE Mutation Does Not Affect Dynamin 2 Oligomerization in the Cell Cytosol

**FIGURE 3:**
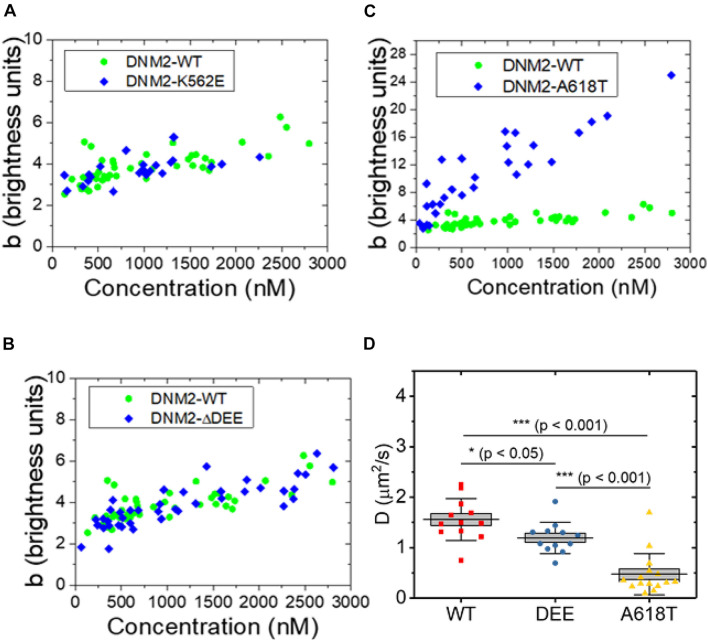
Oligomeric states of WT and mutant DNM2 in the cytosol of U2OS cells. **(A–C)** DNM2^WT^-EGFP (same data plotted in panels **A–C**), DNM2^K562E^-EGFP (panel **A**), DNM2^Δ^ ^DEE^-EGFP (panel **B**), and DNM2^A618T^-EGFP (panel **C**) in the cytosol of U2OS cells as a function of concentration. Monomeric EGFP in U2OS cells was used as the standard. **(D)** RICS analysis of DNM2^WT^-EGFP, DNM2^Δ^ ^DEE^-EGFP, and DNM2^A618T^-EGFP in NIH3T3 cells. Each data point represents the average diffusion coefficient measured in regions of 3.2 μm × 3.2 μm. To compare groups, p-values were calculated with the Mann-Whitney non-parametric test.

We next asked whether the ΔDEE mutation affects the self-assembly of DNM2 in living cells, as it does *in vitro*. We previously reported that DNM2^WT^-EGFP, at concentrations of ∼2 μM or higher, is predominantly tetrameric in the cytosol (James et al., 2014), in agreement with its reported *in vitro* unassembled oligomeric state as measured by analytical ultracentrifugation (Muhlberg et al., 1997). However, the CNM-linked DNM2^*R*369*W*^ mutant forms higher-order cytosolic oligomers, up to 12-14-mers, at concentrations of ∼1 μM or higher (James et al., 2014). Using a refined version of the fluorescence fluctuation spectroscopy (FFS) approach known as brightness analysis (Qian and Elson, 1990; Sanchez-Andres et al., 2005) (see Materials and Methods and Supplementary Figure 1), as in our previous study, we found that EGFP-tagged DNM2^WT^, DNM2^K562E^, and DNM2^Δ^ ^DEE^ are predominantly present in an approximately tetrameric state in the cytosol of living U2OS cells (Figures 3A,B). In contrast, the CNM-linked mutant DNM2^A618T^-EGFP assembled into species as high as 20–24-mers (Figure 3C). Using a distinct FFS approach known as raster scan image correlation spectroscopy (RICS) (Digman et al., 2005), which allows measurement of diffusion in every region of the cell, we obtained average diffusion coefficients (inversely proportional to particle radius) of 1.6 ± 0.4 μm^2^/s, 1.2 ± 0.3 μm^2^/s, and 0.5 ± 0.4 μm^2^/s (mean ± standard deviation) for DNM2^WT^-EGFP, DNM2^Δ^ ^DEE^-EGFP, and DNM2^A618T^-EGFP, respectively (Figure 3D). Although DNM2^Δ^ ^DEE^-EGFP moves somewhat more slowly than DNM2^WT^-EGFP, these results are in general agreement with the N&B data, as a dynamin tetramer has a radius of ∼ 7–8 nm (Muhlberg et al., 1997), whereas dynamin rings (containing 26–28 monomers) are oblate ellipsoids with radii of ∼ 25 nm (Hinshaw and Schmid, 1995). Taken together, our results suggest that the ΔDEE mutation did not induce the formation of high-order DNM2 oligomers in the cytosol, despite its ability to stabilize DNM2 polymers *in vitro*.

### Enhancement of Dynamin 2 Tyrosine Phosphorylation by the ΔDEE Mutation

**FIGURE 4:**
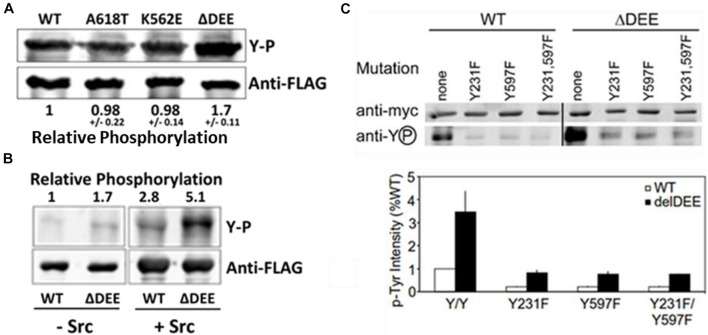
Enhanced tyrosine phosphorylation of DNM2^Δ^ ^DEE^. **(A)**. Phosphorylation in cells. HeLa cells were co-transfected with Src and DNM2s, cell lysates were immunoprecipitated with anti-FLAG Abs and the electrophoresed precipitates were probed with anti-P-Y and anti-FLAG Abs. **(B)**. Phosphorylation *in vitro*. FLAG-tagged DNM2^WT^ and DNM2^Δ^ ^DEE^ were immunoprecipitated from transfected HEK293 cells with anti-FLAG Abs. The beads with immunoprecipitated proteins were treated with alkaline phosphatase (AP, then incubated with or without purified Src. P-Y levels were quantified by LiCOR scanning after blotting with anti-P-Y and anti-FLAG for normalization. **(C)**. Effect of mutations of putative tyrosine-phosphorylated residues. HeLa cells were co-transfected with myc-tagged dynamins and Src. Lysates were immunoprecipitated with anti-myc antibodies and dynamins were pulled down with protein A/G Sepharose. Samples were electrophoresed and blotted with anti-myc and anti-P-Y antibodies.

To date, the most extensively characterized CMT mutant is DNM2^K562E^, which is defective in PIP_2_ binding. Having established that the ΔDEE mutation does not affect the PHD-PIP_2_ interaction, we examined its role in DNM2 tyrosine phosphorylation, which also involves the PHD. Src-mediated tyrosine phosphorylation positively regulates dynamin function in receptor-mediated endocytosis (Ahn et al., 2002; Shajahan et al., 2004; Cao et al., 2010), Golgi budding (Weller et al., 2010), focal adhesion dynamics (Wang et al., 2011), and actin remodeling (Lin et al., 2020). Although the mechanism(s) underlying dynamin regulation by tyrosine phosphorylation are not yet understood, this modification was shown to enhance the self-assembly and GTPase activation of DNM1 *in vitro* (Ahn et al., 2002). We found that DNM2^Δ^ ^DEE^ underwent tyrosine phosphorylation to ∼ double the levels of DNM2^WT^, DNM2^K562E^, or DNM2^A618T^ in cells co-expressing c-Src (Figure 4A) and that DNM2^Δ^ ^DEE^ is phosphorylated to ∼ double the level of DNM2^WT^ by c-Src *in vitro* (Figure 4B). Thus, enhancement of tyrosine phosphorylation is apparently a direct effect of the ΔDEE mutation on the conformation of DNM2.

Two Src-dependent phosphorylation sites have been identified in dynamins, Y231 in the GTPase domain and Y597 in the PHD (Ahn et al., 2002) (see Figure 2B). To test whether increased phosphorylation of these two residues accounts for the hyperphosphorylation of DNM2^Δ^ ^DEE^, we introduced Y231F and Y597F mutations, individually and in combination, into DNM2^WT^ and DNM2^Δ^ ^DEE^ and co-transfected these constructs with c-Src into HeLa cells. As expected, tyrosine phosphorylation of DNM2^WT^ was greatly reduced by introduction of Y231F/Y597F mutations (Figure 4C). Likewise, the Y231F/Y597F mutations diminished tyrosine phosphorylation of DNM2^Δ^ ^DEE^, although to a somewhat lesser extent than occurred with DNM2^WT^.

To determine if the ΔDEE mutation enhances the binding of DNM2 to Src, we compared Förster resonance energy transfer (FRET) between Src-mCherry and DNM2^WT^-EGFP or DNM2^Δ^ ^DEE^-EGFP in NIH-3T3 cells. Binding of Src to dynamin had previously been detected using gel overlay and co-immunoprecipitation assays (Foster-Barber and Bishop, 1998), but the interaction had never been examined in live cells. Using fluorescence lifetime imaging to monitor FRET, we observed that DNM2^WT^ binds to Src in cells (Supplementary Figure 2A) and demonstrated that the ΔDEE mutation had no discernible effect on the binding (Supplementary Figure 2B). As controls, FRET was not observed when DNM2^WT^-EGFP and DNM2^Δ^ ^DEE^-EGFP were expressed in the absence of an acceptor fluorophore (Supplementary Figure 3) or when EGFP and mCherry were co-expressed (Supplementary Figure 4). These results suggest that the increase in tyrosine phosphorylation of DNM2^Δ^ ^DEE^ is apparently not due to an increase in its affinity for the kinase.

## Discussion

At present it is unclear how the ΔDEE mutation confers hypermorphic properties on DNM2. Based on crystal structures of DNM1 dimers, this mutation is not positioned to disrupt the PHD-stalk interface. However, it should be noted that the crystal structure of the DNM3 tetramer failed to reveal the orientation of the two internal PHDs (Reubold et al., 2015). Thus, it is possible that residues _555_DEE_557_ in these internal PHDs contact the stalk or the GTPase domain when not engaged in binding PIP_2_, and that the ΔDEE mutation inhibits these interactions. It is also possible that the ΔDEE mutation in the PHD induces an orientation of the PRD that favors polymerization. Dynamin PRDs are intrinsically disordered ∼120 residue segments that interact with microtubules (MTs) and more than 20 SH3 domain-containing proteins. A subset of monomeric SH3 domains were shown to stimulate DNM1 polymerization and GTPase activation (Krishnan et al., 2015), suggesting an allosteric “de-inhibition” mechanism driven by the PRD. Indeed, deletion of the PRD was found to inhibit DNM1 self-assembly, in contrast to the positive effect on self-assembly induced by deletion of the PHD (Muhlberg et al., 1997). One may envision that the PRD (pI ∼12) interacts electrostatically with the anionic surface of the PHD and that deletion of acidic residues _555_DEE_557_ disrupts that interaction. Functional cross-talk between the PHD and PRD has already been established, as the ΔDEE mutation enhances the PRD-mediated interaction (Herskovits et al., 1993) between DNM2 and microtubules (Tanabe and Takei, 2009, 2012). Moreover, we have shown that the PRD is a critical determinant of dynamin GTPase activation (Barylko et al., 2010).

The functional significance of the enhanced tyrosine phosphorylation of DNM2^Δ^ ^DEE^ is unclear to us at this time. It is unlikely that this modification explains the enhanced *in vitro* stabilization of DNM2^Δ^ ^DEE^ compared to DNM2^WT^ (Figure 2), as the dynamins used in those experiments, which were purified from Sf9 cells in the absence of phosphatase inhibitors, showed similar, very low levels of phosphorylation (Supplementary Figure 5). Because the _555_DEE_557_ deletion affects the PHD, we expected that mutation Y597F would cause a more pronounced decrease in the enhancement of phosphorylation than the Y231F mutation, which affects the GTPase domain. Therefore, we were surprised to find that each mutation had an almost identical effect on the level of phosphorylation, suggesting that deletion of _555_DEE_557_ affects the overall conformation of DNM2, rendering both sites more “phosphorylatable.”

Several lines of evidence support the concept that CNM and CMT are gain- and loss-of-function disorders, respectively. For example, increasing the expression levels of WT DNM2 in mice, either by transfection (Cowling et al., 2011) or by deletion of a microRNA (miR-133A) (Liu N. et al., 2011), induces CNM phenotypes. In contrast, reducing DNM2 expression levels reverses DNM2-linked CNM (Buono et al., 2018), while inducing the CMT phenotype of impaired myelination (Gerber et al., 2019). Likewise, CNM-like histopathological defects are induced by expression of CNM-linked DNM2 mutants in mice (Durieux et al., 2010; Massana Muñoz et al., 2019), Drosophila (Chin et al., 2015), and zebrafish (Gibbs et al., 2014; Zhao et al., 2019). These phenotypes were not induced by expression of CMT-linked mutants (Chin et al., 2015; Massana Muñoz et al., 2019), although expression of the G537C CMT mutant caused slight but significant increases in centralized nuclei in zebrafish muscle (Bragato et al., 2016). Finally, expression of several CMT mutants inhibits clathrin-mediated endocytosis (CME) (Kenniston and Lemmon, 2010; Sidiropoulos et al., 2012; Chin et al., 2015), whereas CNM mutants have little or no effect on CME (Liu Y. W. et al., 2011; Sidiropoulos et al., 2012; Chin et al., 2015); but see conflicting data (Bitoun et al., 2009; Koutsopoulos et al., 2011). Notably, CME was unaffected by expression of DNM2^Δ^ ^DEE^ (Bitoun et al., 2009; Tanabe and Takei, 2009; Liu Y. W. et al., 2011), consistent with our finding that the mutation does not interfere with PIP_2_ binding.

The results of our study do not challenge the currently prevailing view that DNM2-associated CMT is a loss-of-function disease. However, they suggest that more work will be required to identify the distinct physical properties of DNM2 mutants that link these mutants either to CNM or CMT.

## Data Availability Statement

The raw data supporting the conclusions of this article will be made available by the authors, without undue reservation.

## Author Contributions

TT, BB, JM, DJ, RT, and JA planned and designed the study. TT, BB, PH, YC, DB, and NJ performed the experiments. All authors participated in writing and editing the manuscript and approved the submitted version.

## Conflict of Interest

The authors declare that the research was conducted in the absence of any commercial or financial relationships that could be construed as a potential conflict of interest.

## Publisher’s Note

All claims expressed in this article are solely those of the authors and do not necessarily represent those of their affiliated organizations, or those of the publisher, the editors and the reviewers. Any product that may be evaluated in this article, or claim that may be made by its manufacturer, is not guaranteed or endorsed by the publisher.
