# Supplementary material for: Gain-of-Function Properties of a Dynamin 2 Mutant Implicated in Charcot-Marie-Tooth Disease
Source: Front Cell Neurosci. 2021 Oct 20;15:745940. doi: 10.3389/fncel.2021.745940 (PMC8563704; doi:10.3389/fncel.2021.745940)
Supplement: Supplementary file 1 [file Data_Sheet_1.docx]

**Supplementary Information**

**Supplementary Methods**

**Fluorescence lifetime imaging (FLIM).** NIH 3T3 cells were plated on glass-bottom (No 1.5) imaging dishes coated with fibronectin and transfected with plasmids encoding DNM2-EGFP and Src-mCherry using Lipofectamine 3000 (ThermoFisher, Waltham, MA). Cells were imaged 16-20 h after transfection at room temperature for a maximum duration of 90 min with a Zeiss LSM880 (Zeiss, Jena, Germany) laser scanning microscope set up for FLIM. EGFP fluorescence was excited at 880 nm with a 80 MHz pulsed laser (MaiTai, Spectra Physics, Mountain View, CA) via a two-photon process and detected in a band of 510-560 nm with a 40x, NA 1.2 water immersion lens in non-descanned mode using a hybrid photomultiplier detector (HPM-100, Becker & Hickl, Berlin, Germany). The photomultiplier signal was monitored to not exceed 10% of the detector saturation limit. In this regime, the detector response is linear. The photon arrival times were recorded with a fast FLIMBox (ISS, Champaign, IL). For each FLIM data set, 35 frames of 256 x 256 pixels were acquired with a pixel dwell time of 16.38 µs. Data were analyzed using SimFCS software (<https://www.lfd.uci.edu/globals/>). Lifetime data were analyzed using the phasor plot method (Malacrida et al., 2021).

**Supplementary Reference**

Malacrida, L., Ranjit, S., Jameson, D. M., Gratton, E. (2021) The Phasor Plot: A Universal Circle to Advance Fluorescence Lifetime Analysis and Interpretation. *Annu. Rev. Biophysics* 50, 575-593. doi:10.1146/annurev-biophys-062920-063631.

**Supplementary Figure S1.**

**
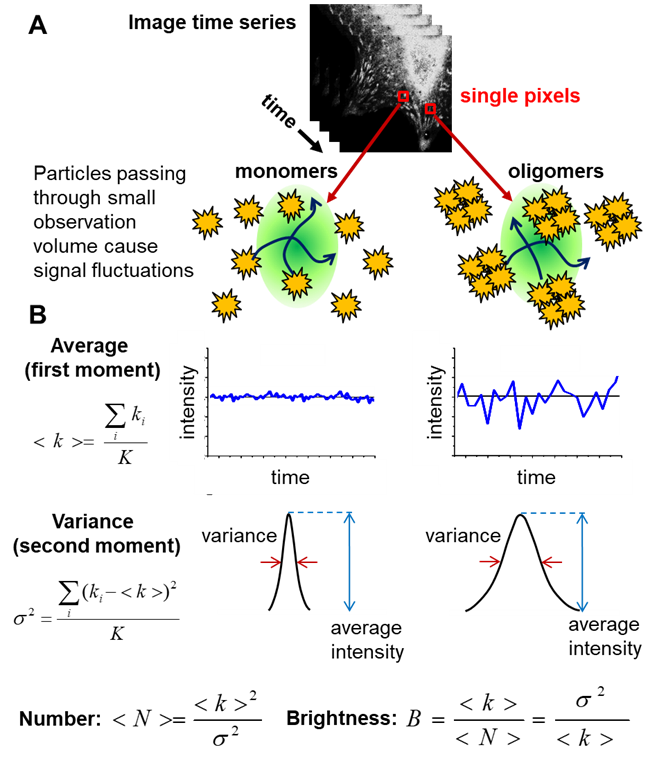
**

**Figure S1. N&B principle.** (A) In each pixel of an image time series, fluorescently labeled particles passing through the observation volume cause intensity fluctuations. (B) As bright oligomers lead to larger signal fluctuations (larger signal variance) than dim monomers, the first (*<k>*) and second moments (*σ^2^*) can be used to calculate the number & brightness (N&B) to map the concentration and oligomeric state of the target molecule in each image pixel.

**Supplementary Figure S2.**

**
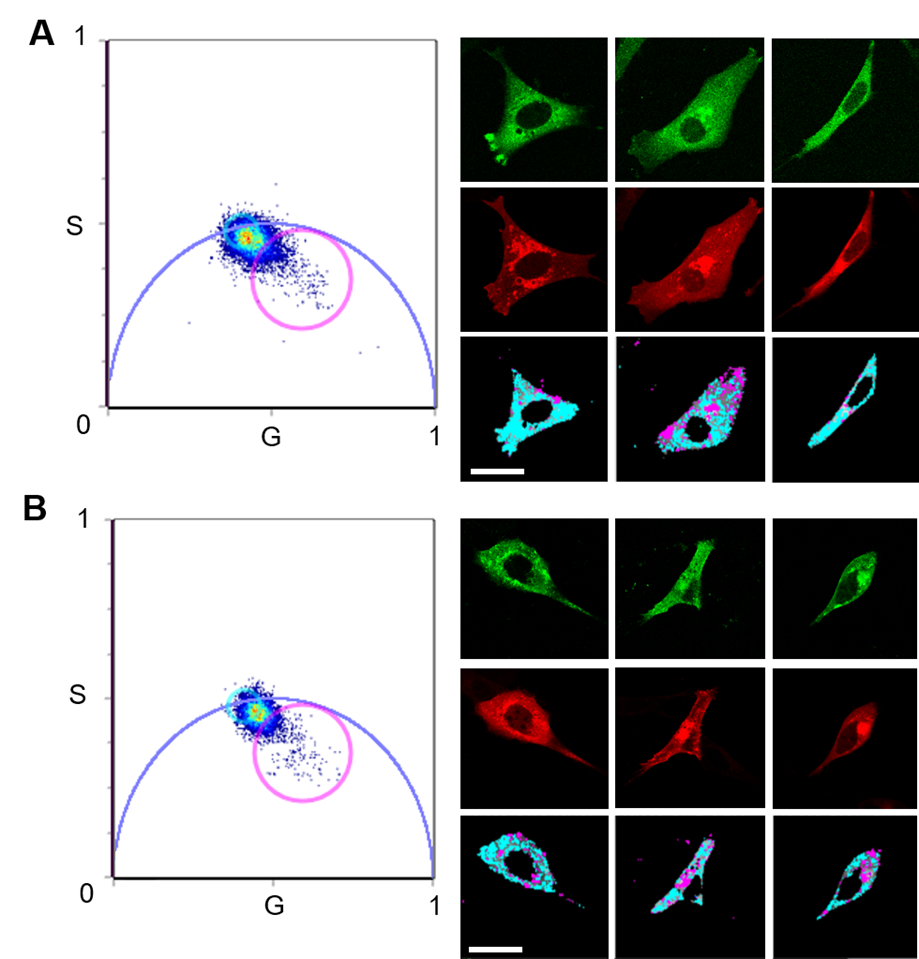
**

**Figure S2. Fluorescence lifetime imaging of NIH-3T3 cells co-expressing EGFP-tagged DNM2s and mCherry-tagged Src.** (A) Cumulative phasor histogram (left) of EGFP fluorescence lifetimes measured for DNM2^WT^-EGFP. Before lifetime imaging, single channel images were taken to determine protein expression levels (right top row, EGFP channel; right middle row, mCherry channel). In the phasor histogram, pixel corresponding to a single exponential decay of 3.4 ns were selected (cyan circle/cursor) and painted in the corresponding intensity images (right bottom row). The presence of shorter, multicomponent lifetimes (selected by magenta circle/cursor) indicates energy transfer from the donor (EGFP) to the acceptor (mCherry) in the highlighted image pixel. (B) Same analysis as in (A) but for DNM2^ΔDEE^-EGFP. Scale bar, 50 µm.

**Supplementary Figure S3.**

**
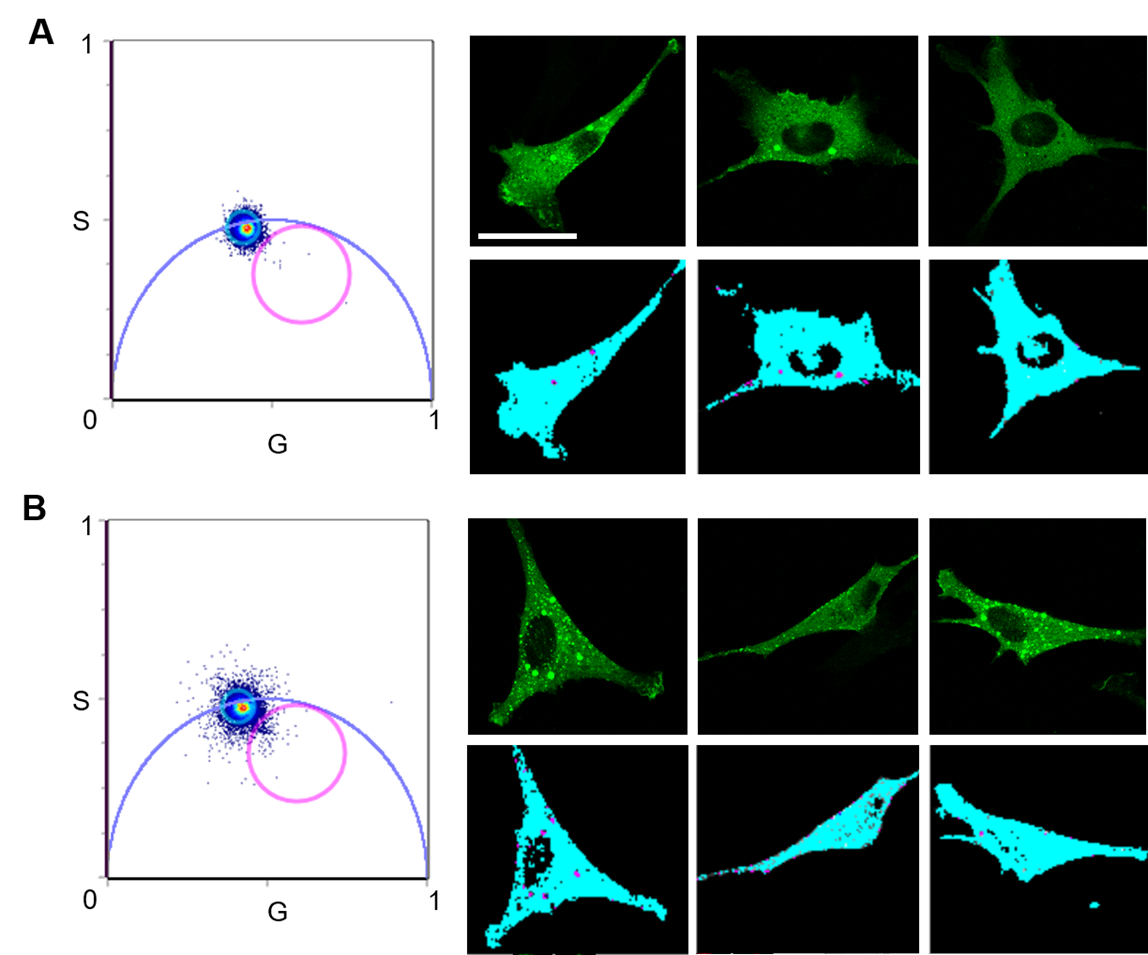
**

**Figure S3.** **Fluorescence lifetime imaging of NIH 3T3 cells expressing DNM2^WT^-EGFP.** (A) Cumulative phasor histogram (left) of EGFP fluorescence lifetimes measured for DNM2^WT^. Before lifetime imaging, single channel images were taken to determine protein expression levels (right top row, EGFP channel). In the phasor histogram, pixel corresponding to a single exponential decay of 3.4 ns were selected (cyan circle/cursor) and painted in the corresponding intensity images (right bottom row). Due to the absence of a donor fluorophore, no shorter, multicomponent lifetimes (selected by magenta circle/cursor) were found. (B) Same analysis as in (A) but for DNM2^ΔDEE^-EGFP. Scale bar, 50 µm.

**Supplementary Figure S4.**

**
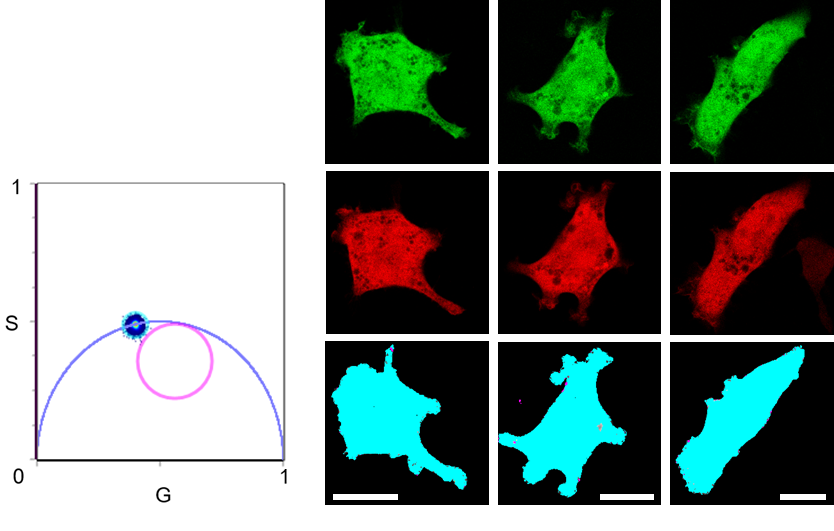
**

**Figure S4. Fluorescence lifetime imaging of EGFP co-expressed with mCherry.** Cumulative phasor histogram (left) of EGFP fluorescence lifetimes. Before lifetime imaging, single channel images were taken to confirm expression of both EGFP (right top row) and mCherry (right middle row). In the phasor histogram, pixels corresponding to a single exponential decay of 3.4 ns were selected (cyan circle/cursor) and painted in the corresponding intensity images (right bottom row). No energy transfer between EGFP and mCherry was detected as no shorter, multicomponent lifetimes (selected by magenta circle/cursor) were found. Scale bars, 20 µm.

**
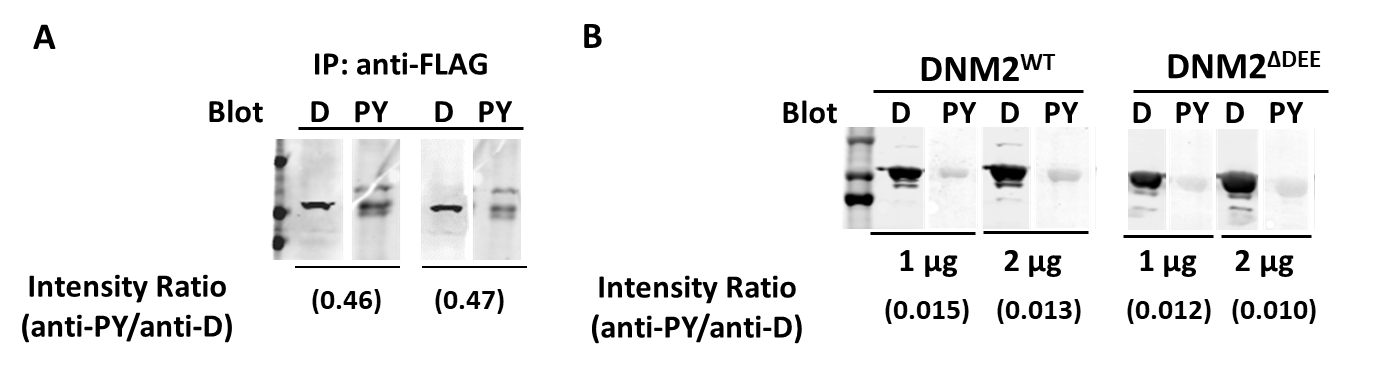
Supplementary Figure S5.**

**Figure S5. Phosphorylation state of DNM2^WT^ and DNM2^ΔDEE^ isolated from mammalian and Sf9 cells.** (A). Phosphorylation of DNM2^ΔDEE^ in HEK293 cells. DNM2^ΔDEE^-FLAG and Src kinase were co-expressed in HEK293 cells and lysates were subjected to immunoprecipitation with anti-FLAG antibodies. Precipitates were electrophoresed and blotted with anti-DNM2 (D) and 4G10 anti-phosphotyrosine (PY) antibodies. Results show duplicate electrophoretic runs of the same sample. (B). Phosphorylation states of DNM2^WT^ and DNM2^ΔDEE^ purified from Sf9 cells as described in the text. As in panel A, samples were electrophoresed and blotted with anti-DNM2 (D) and anti-phosphotyrosine (PY) antibodies. Intensities were quantified by LiCOR scanning for normalization.
